# Fertility-sparing surgery for patients with low-grade endometrial stromal sarcoma

**DOI:** 10.18632/oncotarget.12491

**Published:** 2016-10-06

**Authors:** Weimin Xie, Dongyan Cao, Jiaxin Yang, Xuan Jiang, Keng Shen, Lingya Pan, Huifang Huang, Jinghe Lang, Yan You, Jie Chen

**Affiliations:** ^1^ Department of Obstetrics and Gynecology, Peking Union Medical College Hospital, Chinese Academy of Medical Sciences and Peking Union Medical College, Beijing, China; ^2^ Department of Pathology, Peking Union Medical College Hospital, Chinese Academy of Medical Sciences and Peking Union Medical College, Beijing, China

**Keywords:** fertility-sparing surgery, low-grade, endometrial stromal sarcoma, recurrence, pregnancy

## Abstract

**Purpose:**

To assess the clinical outcomes and fertility of young women with stage I low-grade endometrial stromal sarcoma (ESS) treated with fertility-sparing surgery.

**Results:**

Seventeen patients with stage I low-grade ESS (stage IA, *n* = 6; stage IB, *n* = 11) were entered into this study. Adjuvant hormone therapy was administered to 15 (88.2%) patients. At a median follow-up of 39 months (range, 4106 months), 10 (58.8%) patients developed recurrence. All 10 patients had stage IB disease; among them, the first recurrence limited to the uterus was observed in 6 patients. All 17 patients were alive and disease-free at the time of last contact. After treatment, five of eight (62.5%) patients who attempted pregnancy conceived. No offspring had congenital anomalies.

**Methods:**

Patients with stage I low-grade ESS who underwent fertility-sparing surgery between April 2001 and November 2015 were retrospectively reviewed.

**Conclusions:**

Fertility-sparing surgery may be considered for young patients with stage IA low-grade ESS who wish to preserve their fertility.

## INTRODUCTION

Endometrial stromal sarcomas (ESS) are rare malignant tumors comprising approximately 10%-15% of all uterine sarcomas and 0.2%-1% of all uterine malignancies [1, 2]. The definition and classification of ESS have been modified several times since the initial identification in 1966 [3]. Currently, the 2014 WHO classification divides these malignant tumors into three categories based on pathologic features: low-grade ESS, high-grade ESS, and undifferentiated endometrial sarcoma [4].

Low-grade ESS is the second most common malignant mesenchymal tumor of the uterus [4, 5]; it is more common than the other two types of ESS. Low-grade ESS usually occurs in perimenopausal women but can occasionally occur in young women and adolescents [5, 6]. For patients of childbearing age, fertility preservation is an important factor in the overall quality of life; therefore, this issue has aroused extensive attention. Although fertility-sparing management for other gynecological malignancies has been widely studied, it has been rarely reported for low-grade ESS. Low-grade ESS is a slow-growing malignant neoplasm with an indolent clinical course. Most cases are FIGO stage I disease with a favorable prognosis [1, 7]. Currently, the mainstay of treatment for early stage low-grade ESS is total hysterectomy and bilateral salpingo-oophorectomy (TH/BSO) [5–8]. Because of the rarity of this disease, there are only a few reported cases of fertility-sparing surgery for early stage low-grade ESS [9–20].

Experience is limited and a consensus concerning the safety and effectiveness of fertility-sparing surgery is still lacking. Therefore, we assessed oncological and pregnancy results of young women with stage I low-grade ESS who underwent fertility-sparing surgery.

## RESULTS

A total of 17 patients with stage I low-grade ESS were entered into this study. Table 1 summarizes the main characteristics of patients and tumors. Six patients had stage IA cancers and 11 had stage IB cancers. Median age was 28 years (range, 15-37 years). At the time of surgery, 15 patients were nulliparous and 2 were parous. Progesterone receptor (PR) was positive in all cases, and estrogen receptor (ER) was positive in 15.

**Table 1:** Characteristics of patients (*n* = 17)

Characteristic	No.	%
Age (y), median (range)	28 (15–37)	
Parity		
Parous	2	11.8
Nulliparous	15	88.2
FIGO stage		
IA	6	35.3
IB	11	64.7
Location		
Intramural	12	70.6
Submucosal	5	29.4
Type of initial surgery		
Laparotomy	5	29.4
Laparoscopy	7	41.2
Hysteroscopy	5	29.4
ER		
+	15	88.2
-	2	11.8
PR		
+	17	100
-	0	0
Hormone therapy		
MPA or MA	9	52.9
GnRHa	4	23.5
GnRHa plus LNG-IUD	2	11.8
Not performed	2	11.8
Follow-up (mo), median (range)	39 (4–106)	

Abbreviations: ER, estrogen receptor; PR, progesterone receptor; MPA, medroxyprogesterone acetate; MA, megestrol acetate; GnRHa, gonadotrophin-releasing hormone analogues; LNG-IUD, levonorgestrel-releasing intrauterine device.

Abnormal uterine bleeding (52.9%, 9/17) was the most frequent presenting symptoms. One (5.9%) patient presented with dysmenorrhea. Seven (41.2%) patients were asymptomatic, and two of them reported rapid leiomyoma growth. The preoperative presumptive diagnosis was uterine leiomyoma or adenomyoma for all patients who were definitively diagnosed after the initial surgery. Three patients had simultaneous low-grade ESS and leiomyoma. Five (29.4%) patients underwent laparotomy and seven (41.2%) underwent laparoscopy because the tumors were intramural. Five (29.4%) patients underwent hysteroscopic resection of the tumors that presented as intracavitary polyps. Adjuvant hormone therapy was administered to 15 (88.2%) patients. Of these, 12 received adjuvant hormone therapy immediately after the initial surgery. Three who underwent fertility-sparing surgery at outside hospitals and then experienced intrauterine recurrence received adjuvant hormone therapy after the second fertility-sparing surgery. The most common hormone therapy regimen was medroxyprogesterone acetate (MPA) or megestrol acetate (MA) (52.9%, 9/17). Four (23.5%) patients received gonadotrophin-releasing hormone (GnRH) analogues and two (11.8%) were treated with GnRH analogues followed by levonorgestrel-releasing intrauterine device (LNG-IUD). The remaining two (11.8%) patients received no adjuvant hormone therapy during the period of fertility-sparing management.

At a median follow-up of 39 months (range, 4-106 months), 10 (58.8%) patients developed recurrence over an average period of 17.9 months (range, 3-52 months) (Table 2). All 10 patients had stage IB disease; among them, the first recurrence limited to the uterus was observed in six patients. Of these, four patients underwent a second fertility-sparing surgery and adjuvant hormone therapy. As a result, two patients had a second recurrence and then underwent TH/BSO and two patients were alive without a second recurrence. One patient had recurrence limited to the abdominal wall 24 months after the initial surgery. She underwent local tumorectomy and experienced a second recurrence in the uterus, colon, and abdominal wall 19 months later; she later underwent cytoreductive surgery. Three patients had concurrent intrauterine and extrauterine recurrences. Of these, recurrence was found during pregnancy in two patients who underwent cesarean delivery and cytoreductive surgery followed by adjuvant MA and chemotherapy. All 17 patients were alive and disease-free at the time of last contact.

**Table 2:** Clinical details of patients (*n* = 17)

Case	Age (years)	Stage	Adjuvant therapy	RFS (months)	Location of recurrence	Treatment of recurrence	Location of 2nd recurrence	Attempted pregnancy	Pregnancy	Status (months)
1	15	IA	GnRHa + LNG-IUD	–	–	–	–	No	0	NED (43)
2	33	IA	GnRHa + LNG-IUD	–	–	–	–	No	0	NED (8)
3	34	IA	MPA	–	–	–	–	No	0	NED (4)
4	36	IA	MA	–	–	–	–	Yes	1 (full-term delivery)	NED (38)
5	37	IA	MA	–	–	–	–	Yes	1 (full-term delivery)	NED (24)
6	32	IA	GnRHa	–	–	–	–	Yes	0	NED (39)
7	29	IB	GnRHa	–	–	–	–	Yes	0	NED (55)
8	23	IB	No	21	Uterus	FSS, GnRHa	–	Yes	0	NED (30)
9	28	IB	No	15	Uterus	FSS, MPA	–	Yes	1 (full-term delivery)	NED (54)
10	28	IB	MA	4	Uterus	FSS, MA	Uterus	No	0	NED (39)
11	19	IB	No	7	Uterus	FSS, GnRHa	Uterus, peritoneum	No	0	NED (16)
12	28	IB	MA	3	Uterus	TH/BSO, GnRHa	–	No	0	NED (13)
13	31	IB	MA	18	Uterus	TH/BSO, GnRHa	–	No	0	NED (32)
14	32	IB	MPA	24	Abdominal wall	T	Uterus, colon, abdominal wall	No	0	NED (48)
15	21	IB	MPA	26	Uterus, peritoneum	CS, CRS, MA, CT	–	Yes	1 (preterm delivery)	NED (77)
16	25	IB	No	52	Uterus, abdominal wall	CS+CRS, MA, CT	–	Yes	1 (full-term delivery)	NED (106)
17	26	IB	No	9	Uterus, rectum	CRS, CT, RT	–	No	0	NED (90)

Abbreviations: RFS, recurrence-free survival; FSS, fertility-sparing surgery; TH/BSO, total hysterectomy and bilateral salpingo-oophorectomy; CRS, cytoreductive surgery; MA, megestrol acetate; GnRHa, gonadotrophin-releasing hormone analogues; CT, chemotherapy; RT, radiotherapy; NED, no evidence of disease.

Following treatment, five of eight (62.5%) patients who attempted pregnancy conceived. There were four full-term pregnancies and one preterm pregnancy; all of them were delivered by cesarean section. Four pregnancies were spontaneous and one was achieved by in vitro fertilization and embryo transfer. The mean duration between treatment and pregnancy was 7 months (range, 2-54 months). No offspring had congenital anomalies. Two patients had concurrent intrauterine and extrauterine recurrences that grew quickly during pregnancy and both insisted on continuing the pregnancy. As a result, one of them delivered at 29 weeks and the other had a full-term pregnancy. Both of them underwent cytoreductive surgery immediately after cesarean delivery. One patient became pregnant after the second fertility-sparing surgery and adjuvant hormone therapy. Although it was recommended, no patient underwent prophylactic hysterectomy with or without salpingo-oophorectomy after completion of childbearing.

Previously reported outcomes of fertility-sparing surgery for early stage low-grade ESS are summarized in Table 3. Tumor stage was assessed according to the 2009 FIGO system. To date, only 17 patients with early stage low-grade ESS have been reported. Of these, there were 8 stage IA patients and 7 stage IB patients. One (12.5%) of 8 stage IA patients and 2 (28.6%) of 7 stage IB patients had recurrence during follow-up.

**Table 3:** Results of fertility-sparing surgery for low-grade ESS in the literature

Author (year)	Case	Age (years)	Stage	Adjuvant therapy	Recurrence, months	Pregnancy	Status (months)
Stadsvold (2005)	1	16	IB	MA	–	0	NED (21)
Koskas (2009)	1	34	IA	No	+, 10	1 (NTVD)	NED (23)
Delaney (2012)	1	16	IB	MA	–	1 (C/S)	NED (108)
Sánchez-Ferrer (2012)	1	32	IB	MA	+, 31	1 (Twin pregnancy, C/S)	NED (60)
Choi (2014)	1	31	IA	Letrozole	–	1 (Twin pregnancy, C/S)	NED (99)
Noventa (2014)	1	34	IB	No	–	1 (Pregnant at 11 weeks)	NED (19)
Zhan (2014)	1	26	IB	CT + MPA	–	1 (C/S)	NED (47)
Dong (2014)	1	25	IB	MPA	–	1 (C/S)	NED (31)
Jain (2014)	1	23	IB	No	+, 20	1 (C/S)	NED (54)
Morimoto (2014)	1	25	N/A	MPA	+, 12	0	DOD (> 124)
Maeda (2015)	1	24	N/A	No	+, 10	1 (C/S)	AWD (> 240)
Laurelli (2015)	1	38	IA	No	–	1 (NTVD)	NED (70)
	2	33	IA	MA	–	1 (Spontaneous abortion)	NED (54)
	3	40	IA	MA	–	1 (NTVD)	NED (48)
	4	18	IA	MA	–	0	NED (39)
	5	34	IA	MA	–	0	NED (32)
	6	30	IA	MA	–	0	NED (30)

Abbreviations: N/A, not available; MA, megestrol acetate; MPA, medroxyprogesterone acetate; CT, chemotherapy; NTVD, normal transvaginal delivery; C/S, cesarian section; NED, no evidence of disease; AWD, Alive with disease; DOD, dead of disease.

## DISCUSSION

ESS are rare malignant tumors of endometrial stromal origin that comprise three different subtypes. Low-grade ESS is composed of cells resembling those of proliferative phase endometrial stroma and lack significant cytological atypia or pleomorphism. Unlike high-grade ESS and undifferentiated endometrial sarcoma, which behave aggressively, low-grade ESS has an indolent clinical course with a tendency for late recurrence. For patients with stage I low-grade ESS, 5-year and 10-year survival rates have been estimated to be 98% and 89%, respectively [1], and the median time to recurrence is 65 months [21]. The favorable prognosis and indolent clinical course of stage I low-grade ESS may make it a promising candidate for fertility-sparing surgery.

Low-grade ESS accounts for less than 1% of all uterine malignancies. Patients commonly present with nonspecific symptoms such as abnormal uterine bleeding, abdominal pain, or pelvic pain; however, some are asymptomatic. Because clinical symptoms appear early, most patients initially present with FIGO stage I disease. There are no reliable preoperative imaging modalities or tumor markers that can distinguish low-grade ESS from uterine leiomyoma or adenomyosis [21, 22]; therefore, the preoperative diagnosis of low-grade ESS is difficult.

The standard treatment for stage I low-grade ESS is TH/BSO. Because low-grade ESS is a hormone-sensitive tumor, BSO is recommended for all cases. Recent studies suggest that ovary-sparing surgery does not compromise survival but does have a much higher risk of recurrence [23, 24]. Therefore, it may be considered as an option for premenopausal patients with stage I low-grade ESS. The mean age at diagnosis among 153 patients has been reported as 41.8 years [23]. For young women with stage I low-grade ESS, the pathologic diagnosis is generally made by a conservative resection of a uterine mass that is presumptively diagnosed as uterine leiomyoma or adenomyosis. Needless to say, preservation of reproductive function is a key issue for young women, particularly nulliparous women with low-grade ESS. Several reports have suggested that fertility-sparing management may be a viable option for young women with early stage low-grade ESS [9–20]. Because of the rarity of low-grade ESS, the feasibility and safety of fertility-sparing surgery are still limited.

Although a case involving conservative management of stage III low-grade ESS has been published [25], we focused on the results of fertility-sparing surgery for stage I patients because of the favorable prognosis and relatively longer recurrence-free survival (RFS). In the current study, we reported the clinical courses of 17 stage I low-grade ESS patients (6 stage IA and 11 stage IB) younger than 40 years treated with fertility-sparing surgery. The total recurrence rate was 58.8% (10/17). The 6 stage IA patients had no recurrence and 10 (90.9%) of the 11 stage IB patients experienced recurrence. Another report of 6 stage IA patients treated with fertility-sparing surgery and hormonal therapy also indicated no recurrence [20]. These data suggest that stage IA patients may be candidates for fertility-sparing surgery. Although the recurrence rate for 7 stage IB patients from seven other studies [9, 11, 12, 14–17] was relatively low (28.6%), we observed a higher recurrence rate (90.9%) for our 11 stage IB patients who underwent fertility-sparing surgery. This difference may be partly explained by the relatively short follow-up periods of the previous studies. Among the 10 stage IB patients with recurrence, 6 had recurrence limited to the uterus. Two of four (50%) patients who underwent a second fertility-sparing surgery did not have a second recurrence, and one patient conceived spontaneously and delivered a healthy baby. These results suggest that fertility-sparing surgery for stage IB patients carries a high risk of recurrence; however, recurrence limited to the uterus seems to deserve a second fertility-sparing surgery. Because performing fertility-sparing surgery for stage I low-grade ESS is rare, and because the long-term follow-up results are still lacking, it is not possible to draw any definitive conclusion about its safety.

Most cases of low-grade ESS express ER and PR, which has led to interest in adjuvant hormonal therapy. However, due to its rarity, current data regarding hormonal therapy for low-grade ESS are mainly from case reports and small retrospective series. Data from the literature indicate that adjuvant hormonal therapy may lower the risk of recurrence, but the level of evidence is relatively low. In 2003, Chu et al. [26] reported 22 patients with low-grade ESS; 4 out of 13 (30.8%) who received adjuvant progestins experienced recurrence compared with 6 out of 9 (66.7%) who did not receive hormonal therapy. Hormonal agents include progestins (e.g., MA, MPA), aromatase inhibitors, and GnRH analogues, which are recommended by current international guidelines [27, 28]. However, there is no agreement regarding optimal regimens, doses, or duration of therapy. In our series, patients were mainly treated with adjuvant progestins (MA and MPA) or GnRH analogues and showed good compliance. Because the data were limited, the efficacy of different regimes was not compared. The LNG-IUD was added as maintenance therapy for two patients; no recurrence occurred. These encouraging results indicate that adding the LNG-IUD to progestins or GnRH analogues may be a promising treatment for patients with no plans to conceive in the short term.

The successful pregnancy rate is encouraging; five out of eight (62.5%) patients who tried to conceive became pregnant and delivered successfully. This confirms the results of a previous investigation indicating that three out of six (50%) patients attempting pregnancy conceived [20]. Because most low-grade ESS are sensitive to hormones, there is theoretic potential for tumor growth with increasing circulating hormones during pregnancy [11]. In our series, two patients had concurrent intrauterine and extrauterine recurrences during pregnancy. Therefore, we recommend a complete evaluation to be sure there is no evidence of disease before pregnancy and a strict obstetrical schedule during pregnancy. Because low-grade ESS is characterized by late recurrence, we recommend hysterectomy with or without salpingo-oophorectomy after completion of childbearing, especially for stage IB patients.

To the best of our knowledge, this study represents the largest number of reported outcomes of fertility-sparing surgery for low-grade ESS. Limitations include retrospective data collection, small sample size, and different adjuvant hormonal therapy protocols during the long-term study period. In addition, the long-term oncological outcomes are lacking.

In conclusion, our study suggests that fertility-sparing surgery may be considered for young patients with stage IA low-grade ESS who wish to preserve their fertility. Fertility-sparing surgery for stage IB low-grade ESS carries a high risk of recurrence; however, salvage therapy following local recurrence seems to be effective and does not affect survival outcomes. Patients should be carefully selected and fully informed of the risk of recurrence; they have the right to make their own decisions regarding therapy. Further large-scale studies with long-term follow-up are required to confirm our results and to assess the safety and feasibility of this approach.

## MATERIALS AND METHODS

Patients with stage I low-grade ESS who underwent fertility-sparing surgery at Peking Union Medical College Hospital or those who were referred to the hospital after fertility-sparing surgery performed elsewhere between April 2001 and November 2015 were retrospectively reviewed. Patients were eligible if they had stage I low-grade ESS, if they were treated with fertility-sparing surgery (defined as the resection of the uterine mass and preservation of the uterus and adnexa), and if they were age 40 or younger at the time of fertility-sparing surgery. Patients with insufficient clinical data or who were lost to follow-up immediately after surgery were excluded. Histological diagnosis was established according to the WHO classification, and pathological slides were reviewed by two independent pathologists. Immunohistochemical studies including ER and PR were performed to confirm the diagnosis if necessary. Tumor stage was assessed using the 2009 FIGO system. Stage I was defined as a tumor limited to the uterus; the stage IA tumor size was ≤5cm and the stage IB tumor size was >5cm [8].

The initial surgery was performed via laparotomy, laparoscopy, or hysteroscopy, depending on the location of the uterine mass, which could be submucosal or intramural. When histological results confirmed the diagnosis of low-grade ESS, patients were informed that the standard treatment for stage I low-grade ESS was TH/BSO and that fertility-sparing surgery was only an experimental option. The patients who showed a strong desire to preserve fertility were well informed of the possible risks and benefits of fertility-sparing surgery and signed a consent form. Adjuvant hormone therapy was administered without well-defined protocols. The main hormone therapy included MPA 500 mg/d or MA 160-320 mg/d for 6 months. Alternatively, GnRH analogues administered for 3-6 months were used as another option. Among these patients, two were treated with GnRH analogues followed by insertion of a LNG-IUD. Five patients who underwent fertility-sparing surgery at outside hospitals did not receive adjuvant hormone therapy immediately after the initial surgery.

During hormone therapy, patients underwent transvaginal ultrasonography every 1-3 months and diagnostic imaging or hysteroscopy every 3-6 months. At the end of treatment, all patients underwent follow-up for 3 months during the first year and every 6 months thereafter. Follow-up evaluations consisted of pelvic examination, transvaginal ultrasonography, and periodic diagnostic imaging, including magnetic resonance imagining (MRI), computed tomography (CT), and fluorine-18 fluorodeoxyglucose positron emission tomography/CT (FDG PET/CT). When recurrence confined to the uterus was suggested, patients decided whether to continue fertility-sparing management. If they insisted on preserving fertility, then a second fertility-sparing surgery was performed and adjuvant hormone therapy was administered. If they did not insist on preserving fertility, then TH/BSO was performed. However, cytoreductive surgery was recommended for those exhibiting concurrent intrauterine and extrauterine recurrences. Recurrence rate, RFS, and fertility outcomes were studied. RFS was defined as the time from surgery to the first recurrence.
